# Disease gene prediction for molecularly uncharacterized diseases

**DOI:** 10.1371/journal.pcbi.1007078

**Published:** 2019-07-05

**Authors:** Juan J. Cáceres, Alberto Paccanaro

**Affiliations:** Centre for Systems and Synthetic Biology & Department of Computer Science, Royal Holloway, University of London, Egham, Surrey, United Kingdom; Bar Ilan University, ISRAEL

## Abstract

Network medicine approaches have been largely successful at increasing our knowledge of molecularly characterized diseases. Given a set of disease genes associated with a disease, neighbourhood-based methods and random walkers exploit the interactome allowing the prediction of further genes for that disease. In general, however, diseases with no known molecular basis constitute a challenge. Here we present a novel network approach to prioritize gene-disease associations that is able to also predict genes for diseases with no known molecular basis. Our method, which we have called Cardigan (ChARting DIsease Gene AssociatioNs), uses semi-supervised learning and exploits a measure of similarity between disease phenotypes. We evaluated its performance at predicting genes for both molecularly characterized and uncharacterized diseases in OMIM, using both weighted and binary interactomes, and compared it with state-of-the-art methods. Our tests, which use datasets collected at different points in time to replicate the dynamics of the disease gene discovery process, prove that Cardigan is able to accurately predict disease genes for molecularly uncharacterized diseases. Additionally, standard leave-one-out cross validation tests show how our approach outperforms state-of-the-art methods at predicting genes for molecularly characterized diseases by 14%-65%. Cardigan can also be used for disease module prediction, where it outperforms state-of-the-art methods by 87%-299%.

## Author summary

The elucidation of the genetic causes of diseases is central to understanding the mechanisms of action of a pathology and the development of treatments. Disease gene prediction methods streamline the discovery of the molecular basis for a disease by prioritizing genes for experimental validation. Although some methods use disease phenotype to aid the prioritization, the great majority use outdated static matrices which limits their disease coverage. Our approach uses an updatable disease phenotype similarity, and employs a non-linear transformation to define a prior probability distribution over the genes that mimics the distribution of disease genes in the interactome. Subsequently, a semi-supervised learning method establishes a prioritization ordering for all genes in the interactome, even for diseases with no known molecular basis. Our method can be used not only to obtain a better prioritization for disease-gene associations, but also for retrieving disease modules.

## Introduction

High throughput sequencing and screening techniques have led to an increasing accumulation of genomic data. Despite this growth, the mechanisms of action through which genomic variants drive disease development are not fully understood. As genomic alleles and malignant mutations are continuously sequenced, most of them still miss a functional annotation [1]. Early approaches to find non-experimental disease gene associations were based on linkage analysis, which establishes likelihood of observing alleles in an organism compared to random chance [2]. However, this type of analysis is highly dependent on linkage disequilibrium, and thus traditionally fails on genetically multifactorial and heterogeneous diseases [3]. Alternative approaches, such as genome-wide association studies, do find gene candidates even for complex diseases. However, they often produce hundreds of candidates, making experimental validation expensive and time consuming.

Recent network medicine based approaches bypass the lack of functional annotation by drawing inferences from interaction data. Diseases are seen as perturbations in specific areas of the interactome – the disease modules. Thus the guilt-by-association [4] principle can be applied to find disease genes by prioritizing those close to already known ones. Several approaches have been proposed that exploit this idea and they differ in how they quantify the distance between candidate genes and known disease genes in the interactome. Common measures for the proximity are the number of direct connections, the length of shortest paths and diffusion kernels, including random walkers with restart and propagation flow. For example, Oti *et al*. [5] use direct neighbours, Köhler *et al*. [6] use random walkers with restart, and Navlakha *et al*. [7] include propagation flow and clustering techniques.

Previous authors have also shown that diseases with overlapping modules present significant similarities in terms of phenotype and occurrence (comorbidity) [8]. Phenotypic data has been suggested to be particularly informative as different perturbations in a single disease module often produce similar phenotypes [9, 10], and phenome networks (where genes are nodes that are connected if they show correlated phenotypic profiles) strongly correlate with protein-protein interactions and transcriptional regulatory networks [11]. Furthermore, diseases found in distant neighborhoods in the interactome produce different phenotypes [8]. Several methods have been proposed that combine these different types of data to predict disease genes [12]. One group of methods integrates the data into a unique graph that is then used for the prediction. Lage *et al*. [13] include disease phenotype in the form of clinical features extracted by text mining from scientific papers; Wu *et al*. [14] create binary networks where nodes represent genes, and these are connected when their BLAST E-values is higher than a predefined threshold; Chen *et al*. [15] include information from the Gene Ontology [16], the Mammalian Phenotype [17] and various types of pathway annotations; Li *et al*. [18], Vanunu *et al*. [19] and Mordelet *et al*. [20] include the van Driel disease similarity information [21] to enhance the network; and other authors use heterogeneous networks where nodes can be either diseases or genes – Xie *et al*. [22] connect the nodes with Online Mendelian Disease in Man (OMIM) [23] and MGI mouse phenotype-gene associations, and Zeng et al [24] use HeteSim [25] scores. Another group of methods carries out inferences for each different type of data separately, and then integrate the results. In particular, Aerts *et al*. [26] use co-expression networks, metabolic pathways, Gene Ontology, among others; Franke *et al*. [27] include the Gene Ontology and co-expression networks; Radivojac *et al*. [28] use the Gene Ontology, the Disease Ontology [29], and features based on protein sequence; Karni *et al*. [30] use disease based co-expression networks; and George *et al*. [31] use metabolic pathways and Pfams [32].

Similar techniques have been used on a related problem, that of predicting disease modules – disease genes can then be found within members of these modules. Liu *et al*. [33] recover disease modules through the analysis of gene expression data and partitions of co-expression networks; Ghiassian *et al*. [34] use direct neighbour analysis on protein-protein interaction (PPI) networks to iteratively add genes to the modules.

All these network methods produce high quality results, but require initial seeds (i.e. a few known disease genes) to produce their predictions. In general, results are better when more seeds are available, and several authors have employed disease families (rather than single diseases) which were obtained by aggregating phenotypically similar diseases [5–7], thus increasing the number of initial seeds for their predictions.

An important point to be made here is that there are many molecularly uncharacterized diseases, for which no disease gene is currently known – as of 2018 these comprise 3359 diseases in OMIM, i.e. 39% of the entire OMIM database. For these diseases, most of the methods described earlier are not applicable since the initial seeds are not available (notable exceptions are PRINCE [19] and ProDiGe4 [20], described in the *Methods* section). We shall refer to molecularly uncharacterized diseases as *uncharted*, while those diseases for which at least one disease gene is currently known will be referred to as *charted*.

**Fig 1:**
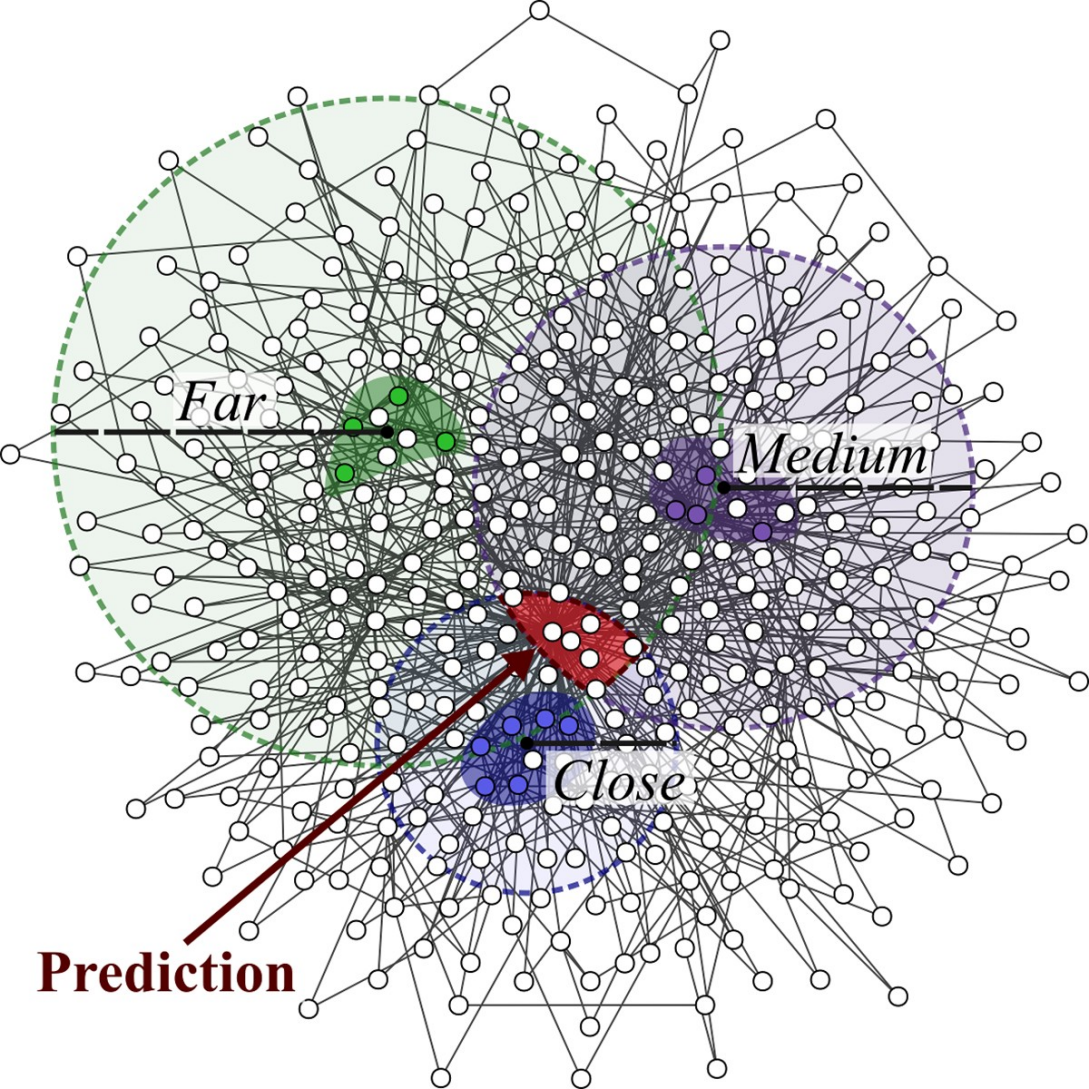
Disease module “triangulation” using disease phenotypic similarity. The area where the module for a disease with no known genes (the query disease, in red) should be located, is identified using the distance to the modules of three charted diseases (blue, purple and green). Colored nodes represent the disease genes of each charted disease and their disease modules are represented with highlighted backgrounds. The distances between the query and the charted diseases (close, medium and far) are represented by the dashed circles and are related to the phenotype similarity (e.g. highly similar diseases should be close in the graph). The disease module for the red disease should lie in the red area.

In this paper, we present a disease gene prediction method that predicts disease genes for both charted and uncharted diseases in OMIM, and can also predict disease modules. Our approach, which we have called Cardigan (ChARting DIsease Gene AssociatioNs), is based on a semi-supervised algorithm that propagates labels on the interactome. These labels integrate disease phenotypic information expressed as a similarity measure between diseases, which is obtained by mining and comparing sets of MeSH terms [35] relevant for the diseases. The approach can be thought of as establishing the location for the modules of charted diseases and using these to “triangulate” the location of the modules of uncharted diseases by exploiting disease phenotypic similarities – the intuition for the approach is shown in Fig 1. We show that Cardigan outperforms state of the art methods in disease gene and disease module prediction.

## Results

### The Cardigan algorithm

Our idea exploits the fact that disease modules of diseases with a similar phenotype should be placed close-by on the interactome [9, 21]. Therefore, genes associated to diseases that are phenotypically similar to a disease of interest should provide useful information to locate its disease module.

**Fig 2:**
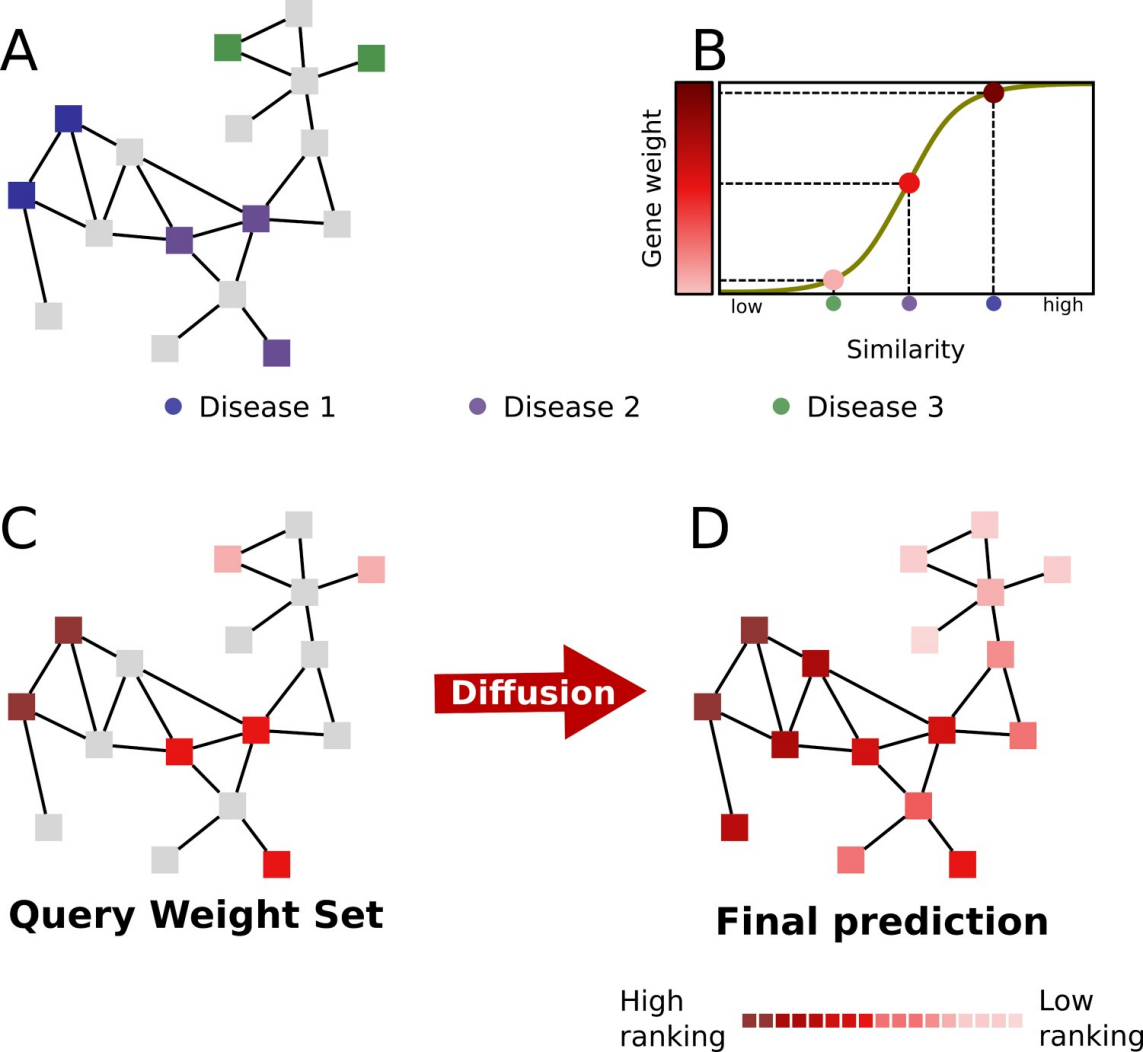
The prediction on an uncharted disease using Cardigan. (A) The PPI network with disease genes associated to three different diseases (green, purple, and blue), is used to predict genes for the uncharted (red) disease. (B) The Caniza similarity is transformed into a weight for the red disease. (C) The *query weight set* (QWS)–the initial seed set for the diffusion process. (D) The final state of the network after the diffusion process. All genes have acquired a weight. These weights are used to rank all genes and constitute Cardigan’s prediction.

To predict disease genes for a given disease (*query disease*), Cardigan begins by calculating its phenotypic similarity to every other disease in OMIM using the approach developed by Caniza *et al*. [36]. Next, Cardigan assigns a weight to each known disease gene. The weight is related to the Caniza similarity between the query disease and the disease to which the gene is associated (Fig 2C). Weights of disease genes are real values between *0* and *1* and are calculated by rescaling the Caniza similarity through a sigmoid function that is dampened by a multiplicative factor 0<*h*<1. (illustrated in Fig 2B; the motivation for the sigmoid function is presented in Section *Significance of the sigmoid* in S1 Text). If a gene is associated with more than one disease, Cardigan uses the highest similarity value. Genes that are already known to be associated with the query disease, if any, are assigned a weight equal to 1 – in this way, these genes are assigned a weight that is higher than the weight of disease genes of any other disease (whose value is at most *h*). For a given query disease, we shall call the set of weights assigned to the disease genes the *Query Weight Set* (QWS) for that disease. The parameters of the sigmoid and the dampening factor *h* were learned using a small training set which we then removed from all subsequent experiments (the training procedure is detailed in Section *Estimation of the default parameters for Cardigan* in S1 Text).

The next step is to propagate the QWS through the graph with a semi-supervised learning procedure (transition between C and D in Fig 2). Cardigan uses the consistency graph diffusion method from Zhou *et al*. [37]. This is a graph labelling procedure based on minimizing a cost function that takes into account network weights and an existing set of labels. Let us represent a weighted PPI network with *n* nodes as an adjacency matrix *W*_*n*×*n*_, where each element *W*_*ij*_ is the weight between genes *i* and *j* (if the network is binary, then all the values in *W* are binary, indicating the presence or the absence of an interaction). The final labelling vector *F* (of size *n*) having one element for each gene, whose value is related to the probability of that gene of being associated with the query disease, is obtained by minimizing the following cost function:

$$C\left(F\right)=\frac{1}{2}\left(\sum_{i,j=1}^{n}W_{ij}{\left\|\frac{1}{D_{ii}}F_{i}-\frac{1}{D_{jj}}F_{j}\right\|}^{2}+\mu \sum_{i=1}^{n}{\left\|F_{i}-Y_{i}\right\|}^{2}\right)$$

where vector *Y* (of size *n*) is the QWS and *μ*>0 is a regularization parameter. Let us briefly analyze the cost function in order to get some intuition for the method (a formal description of the entire procedure is presented in Section *Mathematical formulation of Cardigan* in S1 Text). The cost function being minimized is the sum of two terms. The first term accounts for the consistency of the labels of adjacent nodes (reflecting the *guilt-by-association* principle)–this term is minimized when adjacent nodes have similar labels (i.e. the difference between *F*_*i*_ and *F*_*j*_ becomes small). Also note that the importance of the difference between *F*_*i*_ and *F*_*j*_ is proportional to the edge weight (*W*_*ij*_), i.e. it is related to the probability of the interaction. At the same time, the role of the second term is to conserve the initial labels (QWS), thus it emphasizes the reliability of the initial data for the prediction–this term is minimized when the nodes labels *F*_*i*_ are the same as the initial labels *Y*_*i*_. Finally the *μ* parameter controls the relative importance of the two terms, while the $D_{ii}=\sqrt{\sum_{k=1}^{n}W_{ik}}$ terms serve as normalization parameters for the node degree. The vector *F* that minimizes the above cost function can be interpreted as a gene ranking (Fig 2D), and constitutes the output of Cardigan. The minimum of the cost function above has the following closed form [37]:

$$F=\beta {\left(I-\alpha S\right)}^{-1}Y$$

where *S* = *D*^−1/2^*WD*^−1/2^, $\alpha =\frac{1}{1+\mu }$, and $\beta =\frac{\mu }{1+\mu }$.

It is important here to note that Cardigan is able to predict genes both for charted and uncharted diseases. In fact, the only input for the procedure is the QWS, which can be obtained for both groups of diseases. The only difference is that charted diseases will contain genes with label equal to one corresponding to disease genes already known for those diseases. Furthermore, the method can be used for the prediction of disease modules, since the top predictions of Cardigan can be interpreted as the disease module for the query disease.

### Performance evaluation

We compared the performance of Cardigan against PRINCE, ProDiGe1, ProDiGe4 and DIAMOnD at predicting disease genes for OMIM diseases (these algorithms are described in the *Methods* section). PRINCE and Cardigan were run using both binary protein-protein interaction networks (HPRD [38], BioGRID [39], DiamondNet [34]) as well as weighted networks (HIPPIE [40] and FUNCOUP [41]), while ProDiGe1, ProDiGe4 and DIAMOnD can run only on binary networks (see Methods for details). As a baseline, we also calculated the performance obtained by a procedure that selects disease genes at random. Following previous authors [19, 20, 24], we evaluated the performance at predicting one gene at a time, measuring how often that gene is found within the first 1, 10, 100, 200 genes output by the different algorithms.

We will present the evaluation for charted and uncharted diseases separately, and for each type of disease we will analyze the performance using both time-lapse data and a leave-one-out testing procedure. In time-lapse data experiments, we will attempt to predict genes which have been associated with diseases in the period 2013–17 using data from 2013. Although these experiments are limited in the size of the test set, they are very important as they provide an evaluation of the system in real-life scenarios. In leave-one-out experiments, we will remove a single disease-gene association and measure how well the system can retrieve it.

### Performance on uncharted diseases

**Fig 3:**
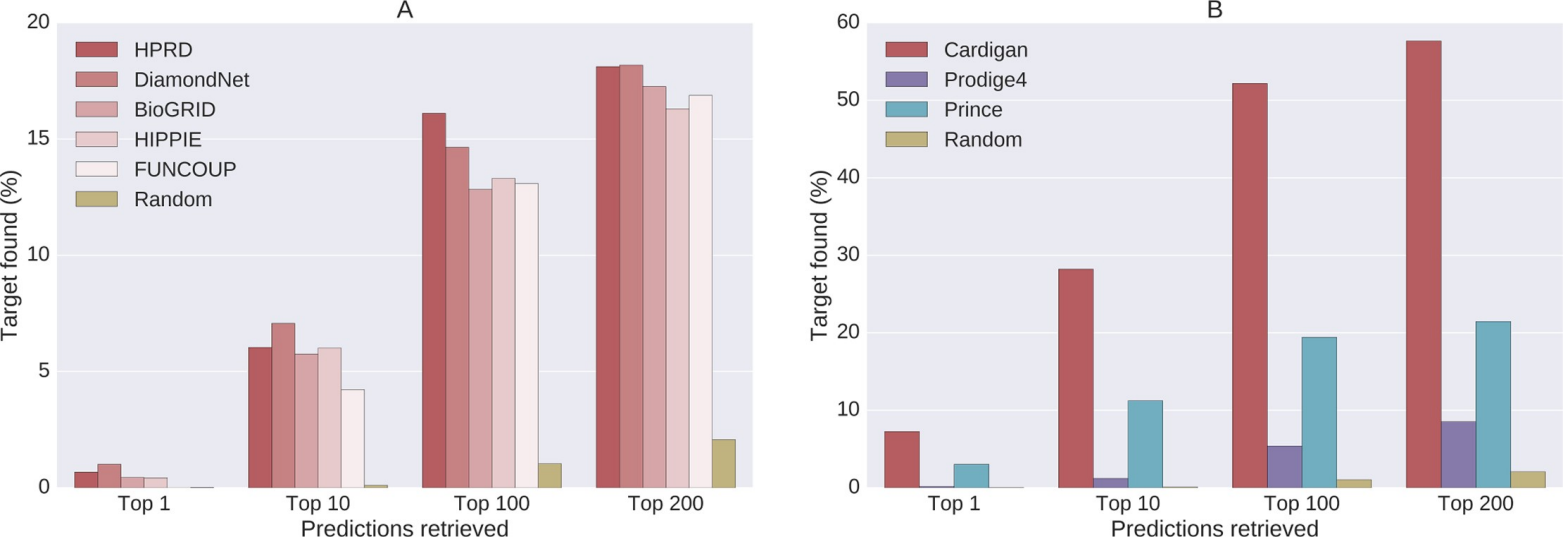
Performance of disease gene prediction for *uncharted* diseases. Percentage of disease genes found in the predictions vs. the number of predictions retrieved. (A) Cardigan performance for diseases which were uncharted in 2013, but were charted in 2017, measured on different PPI networks. (B) Comparison of performances of different disease gene prediction algorithm for a *leave-one-out* testing for diseases with a single known gene in 2017 on HPRD.

**Time-lapse tests:** We begin by presenting the performance of Cardigan at predicting genes that are associated with diseases in 2017, but were uncharted in 2013, using data from 2013. The 2013 OMIM database had 2670 descriptions of uncharted diseases, and 287 of those diseases appear as charted in the 2017 OMIM database. Cardigan is the only method that can make predictions for these 287 diseases. In fact PRINCE and ProDiGe4, the only other methods that could in principle make predictions for uncharted diseases, are not applicable since their disease kernel does not include any of these diseases [21]. The prediction results are presented in Fig 3A, and show that Cardigan has a good performance which is stable across different networks.

**Leave-one-out tests:** If a given disease has only one known disease gene, then by removing it we obtain a “synthetic” uncharted disease. There are 5707 diseases with a single disease gene in the 2017 OMIM database, and for 3252 of them the disease gene were present in HPRD. For each of these diseases we removed its gene and measured the performance of the methods at predicting it back. Since these are synthetic uncharted diseases, there is no initial set of disease genes, and therefore ProDiGe1 and DIAMOnD cannot be used for this problem. Fig 3B shows that Cardigan clearly outperforms both ProDiGe4 and PRINCE for different number of retrieved predictions. Results using the BioGRID, DiamondNet, HIPPIE and FUNCOUP networks were similar and can be found in Section *Other results* in S1 Text.

### Performance on charted diseases

**Fig 4:**
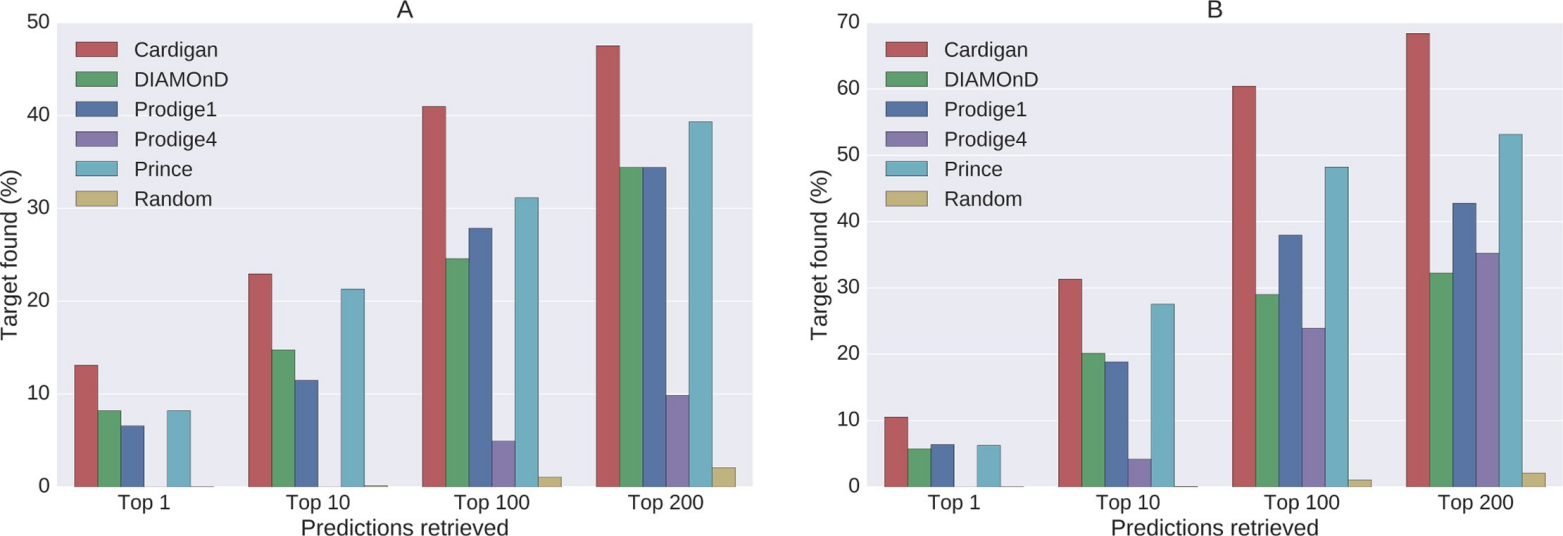
Performance of disease gene prediction for *charted* diseases. Percentage of disease genes found in the predictions vs. the number of predictions retrieved. (A) Performance for predicting genes that charted diseases have acquired between 2013 and 2017. (B) Performances for a *leave-one-out* testing using 2017 data.

**Time-lapse tests:** In these experiments we tested the performance of the different methods at predicting genes for diseases which were already charted in 2013 and gained further genes by 2017, using data from 2013. Out of the 1413 disease gene associations which were new in the 2017 version of OMIM, only 95 of them were added to diseases which were already charted in 2013. This number further reduced for testing since many of these genes were not contained in the PPI networks (their number ranges between 64 for HPRD and 78 for FUNCOUP). Results for HPRD are shown in Fig 4A, where Cardigan presents a minimum improvement of 8% with respect to the second best method at any threshold. Results using the other PPI networks were similar (see Section *Other results* in S1 Text).

**Leave-one-out tests:** This is the typical way in which disease prediction methods are tested [7, 13, 19, 20, 34]. We evaluated the performance of the methods when disease genes were removed one at a time and predicted back. The 2017 OMIM database contains 264 diseases with two or more genes, which result in 970 possible test cases. Fig 4B shows the results for the 826 tests that can be performed using HPRD. We can see how Cardigan outperforms every method at every threshold—the minimum performance improvement is 14% with respect to the second best method at any given threshold. Results using the other PPI networks were similar (see Section *Other results* in S1 Text).

### Performance on disease module detection

We tested how well Cardigan performed at predicting disease modules, i.e. whether the set of predicted disease genes formed a coherent disease module. To do this, we used the same dataset and followed the same procedure that was previously used by Ghiassian *et al*. [34]. Their dataset contains 70 diseases and their respective modules, which had been manually curated. In our experiments, we evaluated the performance of Cardigan at reconstructing the module after removing different percentages of genes (i.e. keeping different percentages of the module). The evaluation measure used is the AUC of the ROC curve normalized for the first 200 false positives predictions, thus matching the sizes of disease modules as described by Ghiassian *et al*. (for more details see Section *Evaluation measure–area under the normalized ROC curve* in S1 Text).

**Fig 5:**
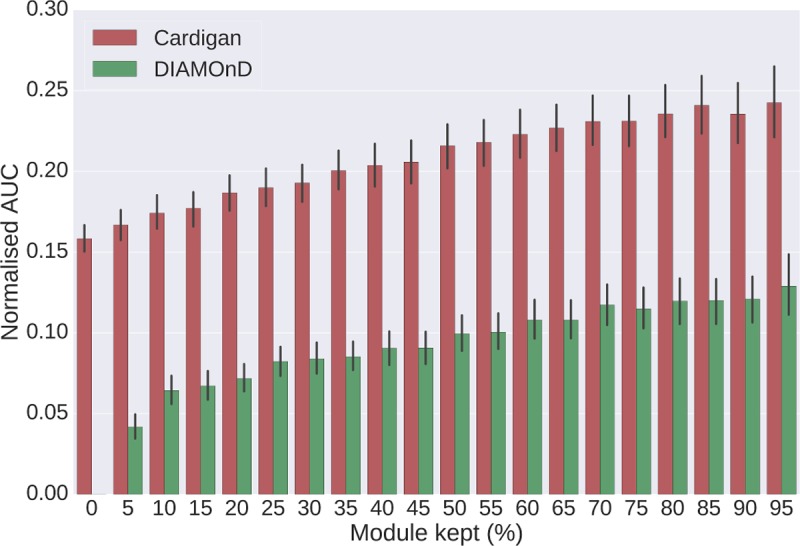
Performance at reconstructing disease modules. Different percentages of disease modules from Ghiassian et al. are removed and modules are then reconstructed. The y-axis shows the AUC of the ROC curve normalized for the first 200 false positives predictions. Error bars were calculated using the results for all diseases, each one with 10 random selections of kept genes. The expected value for a random prediction is 0.007. All predictions were made using DiamondNet.

Fig 5 shows that Cardigan outperforms DIAMOnD consistently when keeping different percentages of the module. At each percentage, we performed 10 random selections of the genes that were kept for each disease to avoid biases on the experiments. The minimum improvement is 87% when 95% of the module is kept, and this goes up to 299% when 5% of the module is kept. Note how Cardigan is also able to recover modules even when 0% of the module is kept. Also, as expected, both methods see an increase in performance as the percentage of kept module increases. We present an additional analysis of the modular properties for the predicted modules of uncharted diseases in Section *Modular properties of sets of predicted genes* in S1 Text.

## Discussion

We have presented Cardigan, a novel network medicine based approach for disease gene prediction. Its key feature is its ability to predict genes for diseases using only their phenotypic description, which allows the method to predict genes for molecularly uncharacterized diseases.

We have shown that Cardigan can handle both weighted and unweighted networks of different sizes by testing it on HPRD, DiamondNet, BioGRID, HIPPIE and FUNCOUP. Our experiments show how Cardigan consistently outperforms by a significant margin state-of-the-art methods and is stable on different types of networks. In particular, Cardigan’s performance remains very high on BioGRID where other methods show significant drops in performance.

The difference in performance between Cardigan and the other methods is larger in time-lapse experiments than in leave-one-out tests, which are more commonly used in the literature. Here we suggest that time-lapse experiments provide a more realistic evaluation as they mimic more closely the gene discovery process. In fact, looking at the evolution of the OMIM database, we notice that genes for complex diseases are frequently discovered (and then added) in groups. The case of adding just one gene at a time, that is portrayed by leave-one-out tests, is much less frequent.

Combining the results over all PPI networks from our time-lapse experiments and considering results among the top 200 genes, Cardigan produces the best gene ranking for 80% of the diseases. Table 1 compiles some interesting examples of Cardigan predictions diseases using the 2013 OMIM database, which were later verified. It includes diseases which had been studied for long periods of time and yet, in 2013, were still missing associated genes–all these diseases have papers in OMIM dated at least from the ‘70s.

**Table 1:** Examples of Cardigan predictions using 2013 data.

*Disease*	*2013 Status*	*Gene*	*Ranking*	*Paper*
*Fetal Akinesia Deformation Sequence (MIM*:*208150)*	Charted	MUSK	1	Tan-Sindhunata et al. (2015) [42]
*Schimmelpenning-Feuerstein-Mims syndrome (MIM*:*163200)*	Charted	NRAS	1	Lim et al. (2014) [43]
*Familial Retinal Arteriolar Tortuosity (MIM*:*180000)*	Uncharted	COL4A1	5	Zenteno et al. (2014) [44]
*Ablepharon-macrostomia syndrome (MIM*:*200110)*	Uncharted	TWIST2	10	Marchegiani et al. (2015) [45]

All the presented diseases appeared in the 2013 OMIM database and already had multiple papers associated with them, describing clinical features, inheritance or molecular genetics. However, in 2013 OMIM did not include the associations with genes shown in the third column, as they first appeared in reference shown in the last column. The position of the gene on the Cardigan predicted ranking is also shown.

An interesting question is whether a QWS (the initial seed set for the diffusion process) can be thought of as an approximate disease module. To verify this, we checked whether its highest ranking genes share functions and whether they tend to be located in the same neighborhood in the interactome. Our analysis shows that genes with higher weights in the QWS for the different diseases are more likely to share function than expected by random, and that the top genes tend to be located in the same neighborhood (detailed description of this analysis is presented in Section *Analysis of modular properties of gene sets* in S1 Text).

Our method differs from earlier kernel methods approaches for scoring disease genes such as, for example, the Lippert *et al*. method [46] which requires a clear distinction between known diseases genes, which are labeled, and other genes, which are unlabeled (more details are provided in Section *Relation between Cardigan and the Lippert method* in S1 Text). In fact, an important difference between Cardigan and other well-known kernelized scoring methods lies in the use of initial labeling for genes other than the known disease genes. Finally, we point out that by including the initial labels, our methodology can be incorporated in a generalized framework, such as, for example, the RANKS tool from Valentini *et al*. [47] (a detailed explanation for RANKS is provided in Section *Generalization of Cardigan as a methodology to include soft labels* in S1 Text).

Finally, the gene rankings obtained by running Cardigan on the entire OMIM diseases set are provided in the S2 Dataset. We believe that this table constitutes an important starting point for the experimental discovery of disease genes, particularly for uncharted diseases.

## Methods

### Disease data

Our experiments were carried out using disease data from the OMIM database [23] downloaded in April 2017. In time-lapse experiments, we also used OMIM data from April 2013 to make predictions which were then verified using the OMIM data from April 2017. Table A in S1 Text summarizes the differences between these two editions of the database.

We also used the Ghiassian et al. [34] diseases module dataset, which encompasses 70 diseases and their modules. These are not necessarily OMIM diseases, and we manually mapped them to OMIM diseases by matching OMIM disease names and taking into account their description. Our mapping from Ghiassian to OMIM diseases is available as a TSV file (S1 Dataset).

### Protein-protein interaction networks

Protein interaction networks come in two flavours, weighted and binary. In weighted networks, links between two proteins are labelled with a weight whose value is related to the probability of the interaction. In binary networks, links are not labelled and a link is either present or missing (denoting the existence or the lack of interaction). Moreover, interaction data can be experimental or predicted. In order to show the general applicability of our methodology, we performed our tests using different types of protein interaction networks including weighted and binary networks with both experimental and predicted data: HPRD [48], DiamondNet [34] and BioGRID [39] are binary experimental networks; HIPPIE [49] is a weighted experimental network; FUNCOUP is a large weighted network including both experimental and predicted data. Table B in S1 Text summarizes some of the relevant characteristics of these networks.

### Other prediction methods

We compared Cardigan to four methods: ProDiGe1, ProDiGe4[20], PRINCE [19] and DIAMOnD [34]. These were chosen because they are state-of-the-art representatives of the disease gene prediction methods and of the disease module prediction methods described earlier.

ProDiGe [20] is a family of kernel-based disease gene prediction methods which rank all genes within the protein-protein interaction network for a given disease. The main idea is to learn missing disease-gene associations through a one-class SVM, where known associations are established as positive labels and the other associations are unlabelled. ProDiGe allows gene associations to be shared among separate diseases. Positive labels are produced by multiplying the known disease-gene association matrix and a disease sharing kernel, and the SVM learns using a graph diffusion kernel created from the PPI network. The four methods in the family (ProDiGe1 to 4) differ in the disease sharing kernel: ProDiGe1 does not share genes (the disease sharing kernel is the identity matrix); ProDiGe2 establishes a uniform low probability to genes from other diseases (the disease sharing kernel is the identity plus a small constant); ProDiGe3 allows genes to be shared by using a phenotype similarity kernel (the disease sharing kernel is the van Driel similarity matrix [21]); and ProDiGe4 adds the kernels from ProDiGe1 and ProDiGe3 to give more importance to the genes of the disease of interest. We chose ProDiGe1 and ProDiGe4 as representatives of the disease gene prediction methods as they have been shown to outperform other well-known methods, such as Endeavour [6], and a multiple kernel learning approach (MKL1class) [20] in the top 200 predictions, and they are comparable in performance to newer methods such as BiRW [22], HSSVM [24] and HSMP [24] when predicting a single disease gene at a time.

PRINCE [19, 50] is a diffusion-based method that uses the Zhou *et al*. iterative propagation [37] to prioritize genes. It makes use of the disease phenotype information provided by the van Driel similarity matrix [21] to gather additional seeds for the query disease. The phenotype information allows genes from highly similar diseases to be effectively regarded as if they were known genes of the query disease (in contrast, our method uses a dampening factor to differentiate the weights assigned to genes from diseases other than the query).

DIAMOnD [34] is a recent disease module prediction method based on direct neighbor analysis which starts from a set of initial seeds and iteratively increases the module by adding new genes. At each iteration, the algorithm evaluates which genes have more connections to the existing disease module than expected by random chance, using the hypergeometric distribution as the null model. The most connected gene according to this model is then added and the authors consider the first 200 to 500 genes as the recovered disease module. Although DIAMOnD is not intended to be a fully-fledged disease gene prediction method, the order in which the genes are added to the module naturally produces a ranking that prioritizes disease genes.

In our experiments, we used the implementations of ProDiGe1, ProDiGe4 and DIAMOnD which were provided in their respective publications. Additionally, we developed our own implementation of PRINCE which uses all the recommended parameters specified in the publication.

### The Caniza similarity

Caniza et al. [36] recently proposed a measure to quantify the phenotypical similarity between hereditary diseases. Their method begins by collecting, for each disease, the set of MeSH terms assigned to the scientific publications relevant for that disease. The phenotype similarity for a pair of diseases is then quantified by the information content of the term on the MeSH ontology that is the lowest common ancestor between the sets of terms for the two diseases. In practice, the similarity is calculated for the diseases found in OMIM, using the publications that OMIM associates to the diseases. The authors have shown that the similarity between two diseases is correlated with the closeness of their respective disease modules on the interactome.

### Implementation

Our method is available as a fast, industrial strength library for Python 2.7 which implements sparse matrices and lazy loading for disease similarities to reduce the memory footprint. The code is publicly available from the paper website at http://www.paccanarolab.org/cardigan.

Although the execution times of the methods are not the main interest of this work, we point out that our method is very fast–a table comparing the execution times of Cardigan with those of DIAMOnD and ProDiGe for the different types of networks can be found in Section *Execution times* in S1 Text.

## Supporting information

S1 Dataset Ghiassian disease dataset to OMIM identifier mapping. The diseases used in the DIAMOnD paper are not necessarily OMIM diseases, so we manually mapped them to OMIM diseases by matching OMIM disease names and taking into account their description.
(ZIP)

S2 Dataset Cardigan prediction on the entire 2017 OMIM dataset. This is a tab separated file containing disease gene predictions for all the diseases with at least one associated paper in the OMIM database.
(ZIP)

S1 Text Supplementary material. This file compiles supplementary definitions and mathematical formulations, model training, description of input data, additional experiments, and a short user manual for our software.
(DOCX)
