# Supplementary material for: Disease gene prediction for molecularly uncharacterized diseases
Source: PLoS Comput Biol. 2019 Jul 5;15(7):e1007078. doi: 10.1371/journal.pcbi.1007078 (PMC6636748; doi:10.1371/journal.pcbi.1007078)
Supplement: S1 Text — This file compiles supplementary definitions and mathematical formulations, model training, description of input data, additional experiments, and a short user manual for our software. (DOCX) [file pcbi.1007078.s003.docx]

**Supplementary Material to**

*Disease gene prediction for molecularly uncharacterized diseases by propagating disease phenotypic similarities*

**Juan J. Cáceres^1^, Alberto Paccanaro^1^**

^1^Department of Computer Science, Centre for Systems and Synthetic Biology, Royal Holloway, University of London, TW20 0EX, Egham, UK.

Correspondence should be addressed to A.P. (alberto.paccanaro@rhul.ac.uk)

# S1 Text

[Mathematical definition of a gene prioritization 3](#_Toc11692565)

[Mathematical formulation of a disease gene prediction instance 3](#_Toc11692566)

[Evaluation measure - average number of targets found in the top predictions 3](#_Toc11692567)

[Building test sets 4](#_Toc11692568)

[Mathematical formulation of a disease module prediction instance 4](#_Toc11692569)

[Evaluation measure - area under the normalized ROC curve 4](#_Toc11692570)

[Mathematical formulation of Cardigan 6](#_Toc11692571)

[Significance of the sigmoid 7](#_Toc11692572)

[Estimation of the default parameters for Cardigan 8](#_Toc11692573)

[Parameters for the QWS 8](#_Toc11692574)

[Parameter for the Diffusion 8](#_Toc11692575)

[Values used in the experiments 8](#_Toc11692576)

[Analysis of the sensitivity of the solution to the choice of parameters 9](#_Toc11692577)

[Description of the interactomes 11](#_Toc11692578)

[Execution times 12](#_Toc11692579)

[Analysis of modular properties of gene sets 12](#_Toc11692580)

[Modular properties of the sets of genes with the highest weights in the QWS 13](#_Toc11692581)

[Modular properties of sets of predicted genes 14](#_Toc11692582)

[Other results 16](#_Toc11692583)

[Results using DiamondNet 16](#_Toc11692584)

[Results using BioGRID 18](#_Toc11692585)

[Results using HIPPIE 19](#_Toc11692586)

[Results using FUNCOUP 21](#_Toc11692587)

[Running the code 23](#_Toc11692588)

[Dependencies 23](#_Toc11692589)

[Installation 23](#_Toc11692590)

[Example 23](#_Toc11692591)

[Relation between Cardigan and the Lippert method 25](#_Toc11692592)

[Generalization of Cardigan as a methodology to include soft labels 26](#_Toc11692593)

[Additional captions 27](#_Toc11692594)

[References 27](#_Toc11692595)

# Supplementary Notes

## Mathematical definition of a gene prioritization

In general, the output of a disease gene prioritization method will be a gene ranking. Predictions can be made for a particular disease with a given set of known disease genes (they can be seen as the query parameters). Most methods will have additional information to produce results, which can be seen as the pool of usable data for any particular prediction.

Formally, let the prediction $P:D\times S\times\partial\to R$, produce a gene ranking $R=\{r_{1},\ldots,r_{k}\}$ for disease $D$ and seed genes $S$, with known data $\partial$. A given method may require different elements within $\partial$, such as gene graphs, gene-gene correlation matrices, extra gene-disease associations, gene expression, or others.

Gene prioritizations will be used for both disease gene and disease module predictions.

## Mathematical formulation of a disease gene prediction instance

A disease gene prediction instance is expected to produce the best possible ranking for a given target gene $t$ in $R$, we denote the prediction function as $P_{t}$, and the ranking $R_{t}$. Naturally, gene $t\notin S$ is restricted to be associated with $D$ in $\partial$.

From this definition, the performance of two predictions $P_{t}$ and $P_{t}^{'}$ are comparable through the position of $t$ in $R_{t}$ and $R_{t}^{'}$ -- i.e. prediction $P_{t}$ is better than $P_{t}^{'}$ if it ranks $t$ closer to the first position, formally: $P_{t}>P_{t}^{'}\to i<j\wedge r_{i}=r_{j}=t$. However, the performance of a set of predictions is established through a statistic measure on the individual predictions. In particular, we calculate how many predictions are found among the top $n$ predictions, and evaluate it with the average number of targets found in the top predictions.

### Evaluation measure - average number of targets found in the top predictions

In this work, we evaluate the performance of a disease prediction method by calculating the fraction of predictions in which the target is found among the top $\tau_{i}$ ranked results. We consider the top $\tau=(1,10,100,200)$ predictions: targets found among the top 1 and top 10 stand as perfect and very high performance in prediction, top 100 represents a good prediction and top 200 is an acceptable prediction (notice that 200 results represent the top 1%-2% genes in the interactomes used).

Formally, for a test set $T=\{(t_{i},D_{i},S_{i})\}$, let the performance of the prediction $P(T)=\{R_{i}\}$ be a vector in $\left[0,1\right]^{k}\in\Re^{k}$, where $k$ is the number elements in $\tau$, defined as:

$$\text{Recall}_{t}=\left(\sum_{j=1}^{n} \frac{1(t_{j},R_{j},\tau_{i})}{n}\right) \forall\tau_{i}$$

where the indicator function $1$ establishes if the target is among the top $\tau_{i}$ ranked genes, and is defined as:

$$1(t,R,\tau)=\left\{\begin{matrix} 1 \text{if} t\in\left\{r_{1},\ldots,r_{\tau}\right\} \\ 0 \text{otherwise} \end{matrix}\right.$$

### Building test sets

The definition of a disease prediction instance requires the existence of a single target to predict per test. This constraint naturally allows the creation of synthetic leave-one-out test sets. However, some diseases gain multiple gene associations over time. Therefore, disease gene prediction of these diseases must split each gene association in a separate prediction instance. Splitting associations as multiple instances have the unfortunate effect of considering the yet unknown associations as negative results; nonetheless, the effect is negligible due to the amount of real non-targets present in the predictions.

*To illustrate, let genes* $a$ *and $b$ be new associations of disease $D$, which need to be predicted. Let $R=\{x,x,a,x,x,x,x,b,x,x,\ldots,x\}$ be the gene ranking obtained for disease $D$ from the information held at the moment of the predictions (where x represents wrong predictions). When* $a$ *is being evaluated, $b$ is regarded as a wrong prediction, but it is not significant, since* $a$ *is ranked higher than $b$. However, when $b$ is being evaluated,* $a$ *is regarded as a wrong prediction, producing an average error of order 1/100 in its ranking. The error is small on its own, and it is further decreased as the test instances are averaged over all the set, and only produces an insignificant lower score in the performance evaluation of a method. Furthermore, as all methods are evaluated with the same measure, the relative performances are not affected.*

## Mathematical formulation of a disease module prediction instance

A disease module prediction instance is expected to produce the best possible ranking for a set of target genes $T=\left\{t_{1},\ldots,t_{m}\right\}\subseteq\left\{r_{1},\ldots,r_{k}\right\}$, we denote the prediction function as $P_{T}$, and the ranking $R_{T}$. Naturally, target genes are not seed genes , and none of them are associated with $D$ in$\partial$.

### Evaluation measure - area under the normalized ROC curve

In this work we evaluate an individual prediction $P_{T}$ considering results only up to the first $X$ false positive results of the ROC curve. We can understand the normalization of the curve, as a “zoom” up to a $\frac{X}{k-m}$ false positive rate in the curve ($k-m$ is the amount of non-targets, i.e. false positives, in $R$), to rescale x-axis a range of $\left[0,1\right]$. Note that the normalized ROC curve does not necessarily reach 1 in the TPR-axis; it will reach 1 if all target genes $T$ are found before $X$ non-targets within the prediction $R$. Furthermore, the random value is not the diagonal of the graph as in the traditional ROC curve; random expectation is as line from $\left(0,0\right)$ to $\left(1,m\frac{x}{k-m}\right)$, which has $\frac{mX}{2\left(k-m\right)}$ AUC.


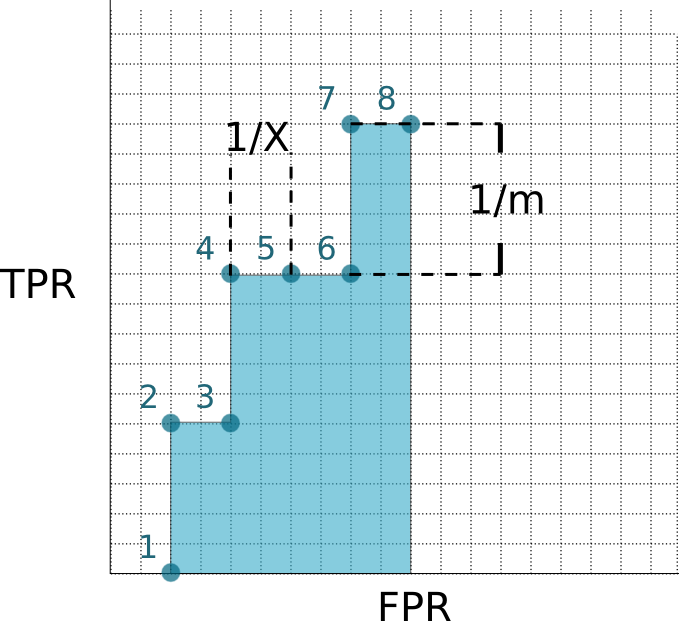


**Fig A. Example of a normalized ROC curve.** In general, there are *m* possible correct results, and we stop collecting results after finding the first *X* negative results. This example shows a prediction in which the first eight predictions are in order: negative, positive, negative, positive, negative, negative, and positive. Note that this figure is partial, and the curve continues until it reaches 1 in the FPR-axis (we are still missing *X*-5 negative results, and possibly some positive results).

Fig A illustrates the curve built as points are added to the prediction. Intuitively, the area under the entire ROC curve is given by calculating the rectangular areas under each different TPR. The FPR axis will be divided in $X$ pieces (the number of false positive retrieved predictions) in the $\left[0,1\right]$ interval, while the TPR axis is divided in $m$pieces (the number of target genes) in the $\left[0,1\right]$ interval.

Note that while it might seem more natural to calculate the bounded ROC measure using the top $X$ predictions instead of the top $X$ false predictions (which can have different lengths, as the number of true positive results can vary). This option could yield misleading results as seen in the following example:

Pretend we are interested in evaluating the ROC curve for a vector with 5 elements and 2 targets. The ideal ranking would be $a=\left[\text{target},\text{target},x,x,x\right]$, while $b=\left[\text{target},x,x,\text{target},x\right]$ would be clearly a worse ranking. The AUC of the ROC curve for $a$ considering only the first two elements would be 0 (there is no step in the FPR axis since there is no false prediction), while the AUC for the ROC curve for $b$ would be 0.5. Under these conditions, a higher measure does not reflect an improvement in the results. The proposal of keeping the top results until reaching the first two false positives gives an AUC of 1 for vector $a$ (a perfect prediction), and 0.5 for vector $b$, which are expected results when comparing both vectors.

## Mathematical formulation of Cardigan

The Cardigan algorithm has two main parts: the construction of the Query Weight Set (QWS) and the diffusion process (which uses the QWS as initial labels).

The QWS for a query disease assigns a weight to all disease genes, and is related to the Caniza similarity between the query and the diseases to which the gene is associated. The gene weights are real values between 0 and 1 obtained by rescaling the Caniza similarity with a sigmoid, effectively classifying the genes as relevant or irrelevant seeds (see Section *Significance of the sigmoid* in S1 Text for more details). Additionally, the outcome of the sigmoid is multiplied by a dampening factor $h<1$ – this is used to differentiate between the known genes (if any) of the query disease (which are clamped to 1) and those of the other diseases, which are at most $h$. If a gene is associated with more than one disease, Cardigan uses the highest weight.

The diffusion process propagates the labels through the graph, balancing a trade-off between the consistency of adjacent labels and the preservation of initial labels. The initial labels are given by the QWS for that disease, and the diffusion uses the consistency method from Zhou *et al.* ^1^.

Formally, the static inputs for Cardigan are an undirected graph $N=\left\langle V,E\right\rangle$ serving as the interactome (represented by an adjacency matrix $W_{n\times n}$), the disease similarity matrix $S:D\times D\to\Re$, and the known disease gene associations $M:D\to G^{+}$. The input parameters are the disease $d\in D$, or list of diseases $d=\left\{d_{1},\ldots,d_{l}\right\}\in D$, i.e. the query, and its known genes $A_{d}:d\to G^{+}$.

For a given prediction, Cardigan collects all the known disease genes from $M$, and assigns the similarity between the query disease and the disease to which the gene is associated in $A'_{d}$. If a gene is associated with more than one disease, Cardigan uses the highest similarity value. Formally:

Then, $A_{d}$ and $A'_{d}$ are converted into the Query Weight Set (QWS) used for the diffusion vector $Y$, defined as:

where $a$ and $b$ are the center, slope of the sigmoid, and $h$ is the dampening factor –i.e. the maximum weight attainable by genes not associated to the query.

The information from the QWS serves as an initial labelling vector, which is then propagated through the interactome, is held in vector $Y$. The final labels of the diffusion vector $F$ are those which minimize the cost function $C(F)$, i.e. $F=\mathrm{argmin}\left\{C(F)\right\}$. The cost function is defined as:

$$C\left(F\right)=\frac{1}{2}\left(\sum_{i,j=1}^{n} W_{ij}\left\|\frac{1}{\sqrt{\sum_{k=1}^{n} W_{ik}}}F_{i}-\frac{1}{\sqrt{\sum_{k=1}^{n} W_{kj}}}F_{j}\right\|^{2}+\mu\sum_{i=1}^{n} \left\|F_{i}-Y_{i}\right\|^{2}\right)$$

The vector $F$ that minimizes the cost function has the following closed form:

$$F=\beta\left(I-\alpha S\right)^{-1}Y$$

where $S=D^{-1/2}WD^{-1/2}$ is calculated from the input graph $W$, and the scalars $\alpha=\frac{1}{1+\mu}$, and $\beta=\frac{\mu}{1+\mu}$depend on the regularization parameter $\mu$. The resulting vector $R=\left\{r_{1},\ldots,r_{n}\right\}$ is produced by sorting all genes in the graph by their final label $F=\left\{f_{1},\ldots,f_{n}\right\}$ in decreasing order. Note that all genes in the graph are in the prediction, i.e. $\bigcup_{i=1}^{n} r_{i} =V$.

### Significance of the sigmoid

The sigmoid is intrinsically related to a two-class classification problem which arises when deciding whether a gene is a seed disease gene (class $C_{1}$) or not (class $C_{2}$) based on the similarity value. Bayes theorem defines an identity of conditional probabilities:

$$P(A|B)=\frac{P(B|A)P(A)}{P(B)}$$

Given that a similarity value $x$ can only belong to one of the two classes $C_{1}$ or $C_{2}$, the law of total probability states that:

$$P(x)=P(x|C_{1})P(C_{1})+P(x|C_{2})P(C_{2})$$

Therefore, the posterior probability of a class given a similarity value $x$can be written as:

$$P(C_{i}|x)=\frac{P(x|C_{i})P(C_{i})}{P(x|C_{1})P(C_{1})+P(x|C_{2})P(C_{2})}$$

We can simplify the equation by calculating the log odds between the classes (without loss of generality, we present the solution for $C_{1}$):

$$r=\ln\frac{P(x|C_{1})P(C_{1})}{P(x|C_{2})P(C_{2})}$$

Replacing $r$ in the posterior gives:

$$P(C_{1}|x)=\frac{1}{1+\exp(-r)}=\sigma(r)$$

which is the sigmoid function^2^.

Therefore by choosing the sigmoid function, given a query disease, the value assigned to each gene in its QWS can be interpreted as the posterior probability for that gene to be a disease gene for the query.

## Estimation of the default parameters for Cardigan

Our method has four parameters: three for the defining the QWS and one for the diffusion. These parameters were all tuned using one disease: Acute Lymphoblastic Leukemia (MIM:613065) that has 8 known disease genes. The tuning was done by changing the parameters to optimize the performance on prediction tests in which a single known disease gene was kept, while the other 7 were removed and used as targets. The performance was evaluated by the average position of the 7 target genes. Importantly, Acute Lymphoblastic Leukemia (MIM:613065) was never again used in any of our tests in the manuscript. Below we give details of the tuning procedure.

### Parameters for the QWS

Therefore, the parameters defining the QWS are the dampening factor $h$ and slope $a$ and center$b$ of the sigmoid. The dampening factor $h$ was screened between 0.05 and 1 on intervals of 0.01. Rather than tuning the values of the slope and center of the sigmoid directly, we defined two similarity values, which we shall call $s_{10}$ and $s_{90}$ such that $\sigma_{a,b}(s_{10})=0.1$ and $\sigma_{a,b}(s_{90})=0.9$. We tested all combinations of $0<s_{10}<s_{90}<5$ in steps of 0.1.

### Parameter for the Diffusion

The Zhou method contains a single parameter to tune, $\alpha=\frac{1}{1+\mu}$. The parameter was tested from $0.01$ to $0.99$ in intervals of $0.01$.

### Values used in the experiments

In our experiments, we used the following parameters: for the binary networks HPRD, BioGRID and DiamondNet are $h=0.50$, $s_{90}=3.0$, $s_{90}=4.0$, and $\alpha=0.69$; while the weighted networks FUNCOUP and HIPPIE use $h=0.60$, $s_{90}=3.0$,$s_{90}=4.0$, and $\alpha=0.75$.

### Analysis of the sensitivity of the solution to the choice of parameters

Our method is not very sensitive to the choice of the regularization parameter. To show this, we evaluated the performance of our method using the leave-one-out charted test set on HPRD (which contains 769 different disease-gene associations) for values of the sensitivity parameter $\alpha$ ranging from 0.0 to 0.99 in steps of 0.01. The results are plotted in the figure below.


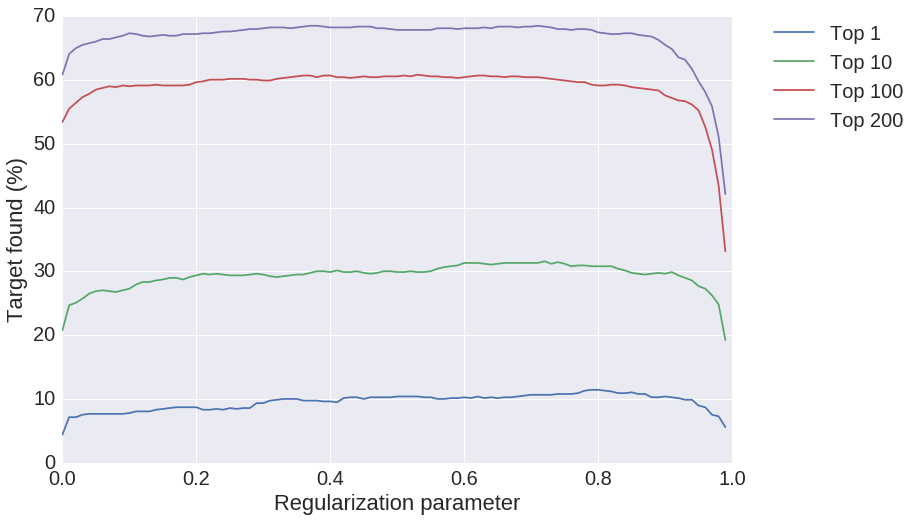


**Fig B. Sensitivity to the regularization parameter.** Each line shows the performance of Cardigan for a particular recall size as the regularization parameter varies in steps of 0.01, from 0.0 to 0.99. The experiment was performed for the entire leave-one-out charted test set on HPRD.

Fig B shows that there is a wide interval for the regularization parameter in which the performance is adequate – mostly between 0.2 and 0.9.

Our method is more sensitive to the choice of the sigmoid parameters. This is to be expected as it controls the initial seeds of the diffusion process. With the parameters of the sigmoid that we used in our experiments, the QWS are quite “conservative”: for a given query disease its QWS contains only very few genes with a high value transferred from other diseases. This can be clearly seen in Fig C below.


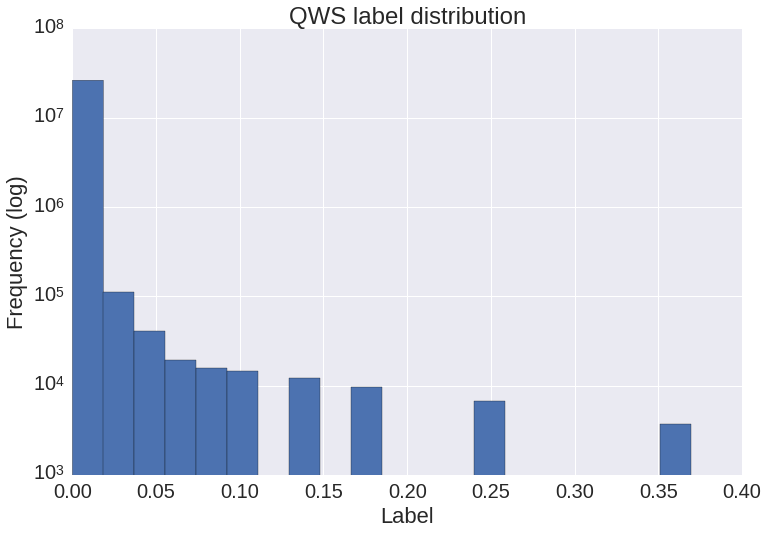


**Fig C. Histogram of the QWS weights for all charted diseases.** The y-axis represents the frequency of the weights. The x-axis represents the different weights obtained from diseases other than the query. These counts are made on an aggregate of 5708 charted diseases for the 9670 genes in HPRD.

These high labels will dominate the diffusion process. As long as the parameters of the sigmoid are set on a similar way, and only very few highly similar disease genes are given a high seed value, the diffusion process will produce reasonable results. This is to be expected, as providing high labels for remotely related diseases genes amounts to providing incorrect information to the procedure.

## Description of the interactomes

We compiled some relevant facts about the networks used and disease gene associations, in order to give a sense of possible recall rates for the different graphs in the paper.

| **OMIM Release** | **# of Charted Diseases** | **# Charted Diseases with 2 or more known genes** | **# of Uncharted Diseases** | **# of Different Genes** | **# of Disease Gene Associations** |
| --- | --- | --- | --- | --- | --- |
| **2013** | 4870 | 293 | 2670 | 4040 | 6303 |
| **2017** | 5992 | 264 | 2388 | 4820 | 7292 |

**Table A: Relevant counts from the OMIM Databases.** The number of charted diseases includes diseases with 2 or more known genes. The number of uncharted diseases accounts for all diseases with no known molecular basis which have annotated publications (diseases with annotated suspected genes are not included in this count). The number of different genes is the set of all disease genes (some genes belong to multiple diseases). The number of disease gene associations are all unique disease-gene pairs annotated in the OMIM database.

Notice that while the 2017 OMIM database includes 989 more disease associations than the 2013 release, over 1400 associations are not included in the older version. This difference comes from changes in diseases identifiers between versions, changes in the genes associated to the diseases, and even removed diseases.

| **Network** | **Nodes** | **Edges** | **Coverage OMIM2013** | **Coverage OMIM2017** | **Edge Type** |
| --- | --- | --- | --- | --- | --- |
| **HPRD** | 9670 | 39220 | 54% | 54% | Exp. Binary |
| **DiamondNet** | 13460 | 141296 | 65% | 66% | Exp. + Inf. Binary |
| **BioGRID** | 19803 | 279187 | 69% | 71% | Exp. Binary |
| **HIPPIE** | 16552 | 239684 | 71% | 74% | Exp. Weighted |
| **FUNCOUP** | 18113 | 4476818 | 71% | 74% | Exp. + Inf. Weighted |

**Table B: Characteristics of Protein-Protein interaction networks.** Coverage shows the fraction of the different disease genes from the OMIM database found in the network (see Table A). Edge type shows the type of evidence (exp. for experimental, inf. for inferred), and whether the edges are binary or weighted.

Note that even if a gene is found in a network some methods can still fail to predict it. Both ProDiGe and DIAMOnD in practice require that the predicted genes are located in the main connected component of the network[[1]](#footnote-1). However, Cardigan is still able to predict genes within the other components of the network if there is a known disease gene in said component.

## Execution times

Although time is not an essential aspect for disease gene prediction, the run time of the used turn appear to vary considerably. We produced a table with an average prediction time for each algorithm using different networks. Note that these measurements do not include the initial time to load data into memory for any of the methods.

| **Network** | **Cardigan** | **DIAMOnD** | **Prodige 1** | **Prodige 4** |
| --- | --- | --- | --- | --- |
| **HPRD** | 0.48 | 1.32 | 2.59 | 176 |
| **DiamondNet** | 0.62 | 3.86 | 4.79 | 291 |
| **BioGRID** | 0.92 | 7.73 | 9.98 | 433 |
| **HIPPIE** | 0.75 | - | - | - |
| **FUNCOUP** | 1.10 | - | - | - |

**Table C: Average run times in seconds for a single prediction on different interactomes.** The averages were taken from the same test set with over 100 predictions on the same system. Intel XEON 2.6GHz, 32 GB RAM running Debian Jessie.

Notably, all the presented methods except DIAMOnD produce a ranking for all genes in the network. DIAMOnD is only producing 200 predictions, as the iterative nature of the method allows the procedure to stop when a certain number of results is produced.

## Analysis of modular properties of gene sets

We analyzed to what extent, for uncharted diseases, the genes with the highest weights in the QWS and the top predicted genes constitute a module. This was done by checking whether these genes share functions and whether they tend to be located in the same neighborhood on the interactome.

Recall that the Cardigan algorithm (*Results* Section from the main manuscript) starts by establishing a set of weights for all known disease genes based on the phenotype similarity between the query disease and the disease to which the gene is associated – we have called this the Query Weight Set (QWS) for a disease. The QWS is then propagated through the graph to produce the final weight for every node in the network. These final weights are used to rank all genes and constitute Cardigan’s prediction for the query disease. In the following, we shall continue to refer to the weights which serve as input for the diffusion process as the *QWS*. Likewise, we refer to the prediction of the method as the *output* weights.

For each of the 2388 uncharted diseases, we created sets of genes with the highest 10, 100 and 200 values in the QWS (analysis of *sets of genes with the highest weights in the QWS*), and sets of genes with the top 10, 100 and 200 output weights (analysis of *sets of predicted genes*). Experiments were performed using the largest connected component (LCC) of HPRD. For each set of genes, we measured the Module Homogeneity, as well as the average distance between each pair of genes in the set (i.e. the average length of the shortest path between them). The idea of measuring the Module Homogeneity was taken from Goh *et al.* ^3^ and it consists in calculating, for each gene set, the fraction of genes annotated with the most popular function – here we used all three GO ontologies (Molecular Function, Biological Process and Cellular Component) while excluding the less informative terms appearing on the top 3 levels of the ontologies. We also calculated the same quantities for 2388 sets of genes of size of 10, 100 and 200 chosen at random. The following subsections present the results of our analysis where we compared these quantities for the QWS sets and the output sets vs the random sets.

### Modular properties of the sets of genes with the highest weights in the QWS


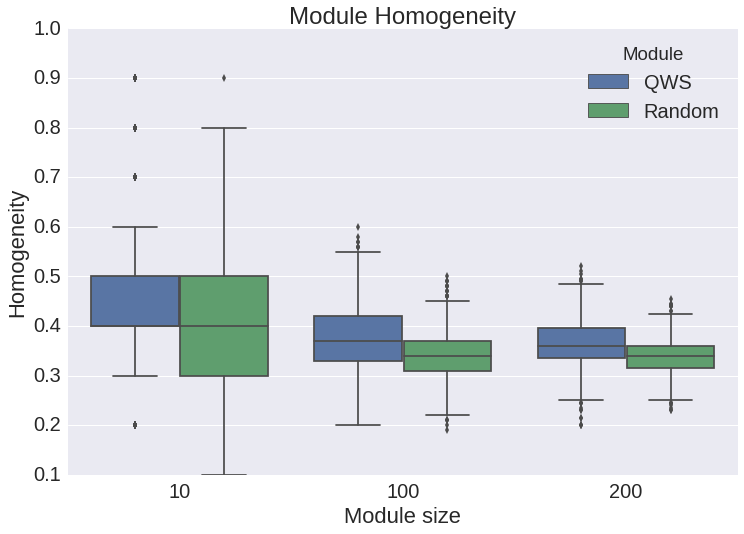


**Fig D. Module Homogeneity.** Comparison of the most popular GO term annotation for sets of highest-ranking genes in the QWS (blue) and random sets of genes (green). Results are shown separately for gene sets of different sizes (10, 100, 200). A higher value indicates a more functionally homogeneous set.


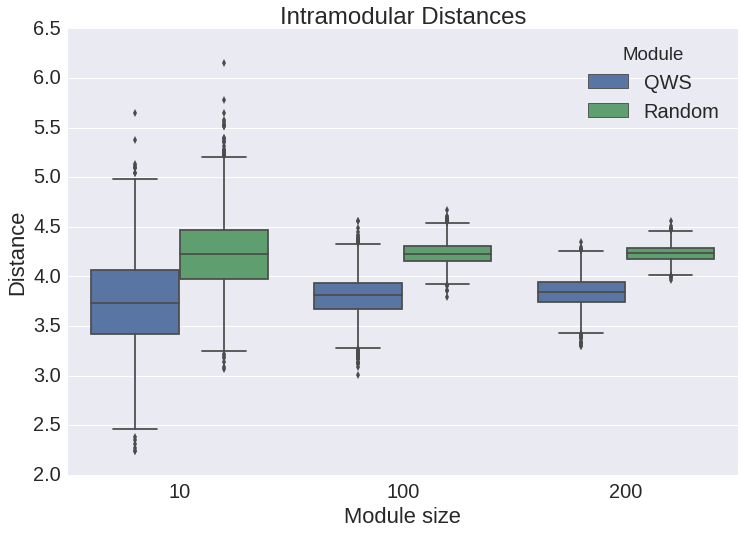


**Fig E. Average intra-modular distances.** Comparison of the average intra-modular distance for sets of highest-ranking genes in the QWS (blue) and random sets of genes (green). Distances were calculated on HPRD. A lower value indicates a tighter set of genes.

Comparing sets of highest-ranking genes in the QWS with random sets of genes informs us about the modular properties of the QWS sets. Figs D and E show that, for uncharted diseases, sets of genes containing the 10, 100 and 200 highest ranking genes in the QWS are more likely to share function and are located closer on the interactome than expected at random. The difference is statistically significant, as shown by the p‑values, calculated with the non-parametric Mann-Whitney U‑Test, shown in the Table D, below:

| Subset | Homogeneity p-value | Avg. Distances p-value |
| --- | --- | --- |
| Top 10 | 7.19e-55 | 1.90e-252 |
| Top 100 | 2.37e-88 | < 1.0e-308 |
| Top 200 | 4.24e-89 | < 1.0e-308 |

**Table D: Significance of the difference between the 10, 100 and 200 highest ranking genes in the QWS and random subsets of genes of the same size.** p‑values were calculated with the non-parametric Mann-Whitney U‑Test. A low p-value indicates that one distribution is stochastically greater than the other.

### Modular properties of sets of predicted genes


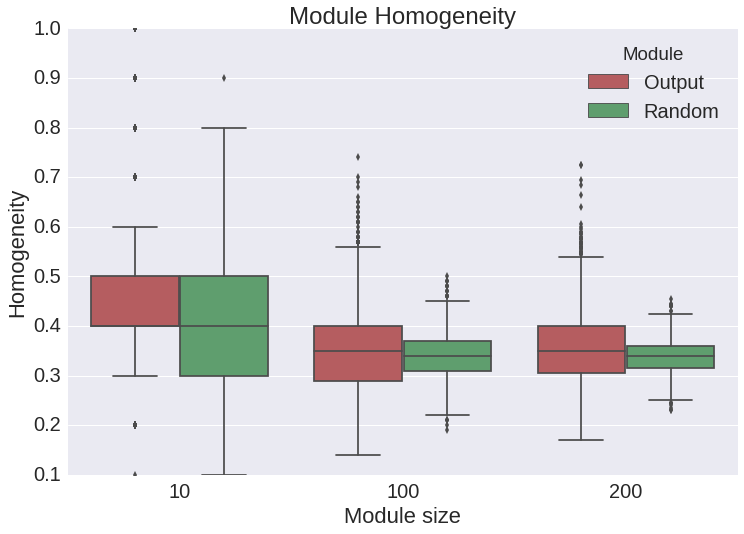


**Fig F. Module Homogeneity.** Comparison of the most popular GO term annotation for sets of top ranking predicted genes (red) and random sets of genes (green). Results are shown separately for gene sets of different sizes (10, 100, 200). A higher value indicates a more functionally homogeneous set.


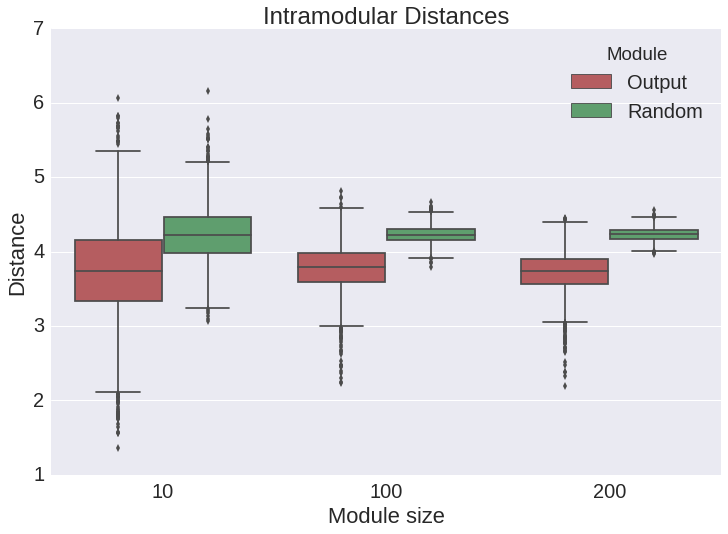


**Fig G. Average intra-modular distances.** Comparison of the average intra-modular distance for sets of top ranking predicted genes (red) and random sets of genes (green). Distances were calculated on HPRD. A lower value indicates a tighter set of genes.

Comparing sets of top predicted genes and random sets of genes informs us about the modular properties of Cardigan’s output**.** Figs F and G show that, for uncharted diseases, the sets of the top 10, 100 and 200 genes output by Cardigan are more likely to share function and are located closer on the interactome than expected at random. The difference is statistically significant, as shown by the p‑values, calculated with the non-parametric Mann-Whitney U‑Test, shown in the Table E, below:

| Subset | Homogeneity p-value | Avg. Distances p-value |
| --- | --- | --- |
| Top 10 | 2.70e-24 | 7.31e-193 |
| Top 100 | 4.58e-31 | < 1.0e-308 |
| Top 200 | 6.79e-63 | < 1.0e-308 |

**Table E: Significance of the difference between top 10, 100 and 200 genes output by Cardigan and random subsets of genes of the same size.** p‑values were calculated with the non-parametric Mann-Whitney U‑Test. A low p-value indicates that one distribution is stochastically greater than the other.

## Other results

We include the results on different combinations of networks and OMIM databases to make an extensive observation of the results presented in the main paper. As the implementations provided by ProDiGe1, ProDiGe4 and DIAMOnD can only run on binary networks, tests are produced for HPRD (in the main paper), DiamondNet and BioGRID.

Since only Cardigan could produce results for time-lapse uncharted diseases (ProDiGe4 and PRINCE, the only other methods that could in principle make predictions for uncharted diseases, are not applicable since their disease kernel does not include any of these diseases), we show the comparison for time-lapse charted, leave-one-out charted and leave-one-out uncharted experiments.

### Results using DiamondNet


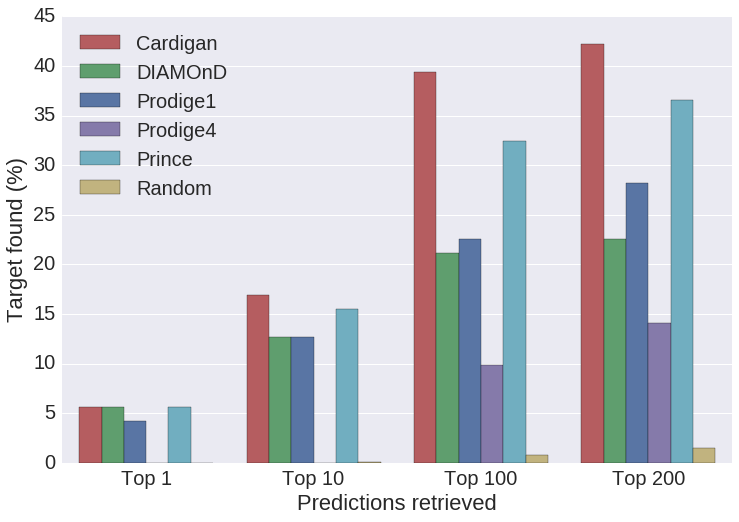


**Fig H. Time Lapse Charted using DiamondNet.** Out of the 1413 disease gene associations which were new in the 2017 version of OMIM, only 95 of them were added to diseases which were already charted in 2013, of which 71 can be predicted using DiamondNet.


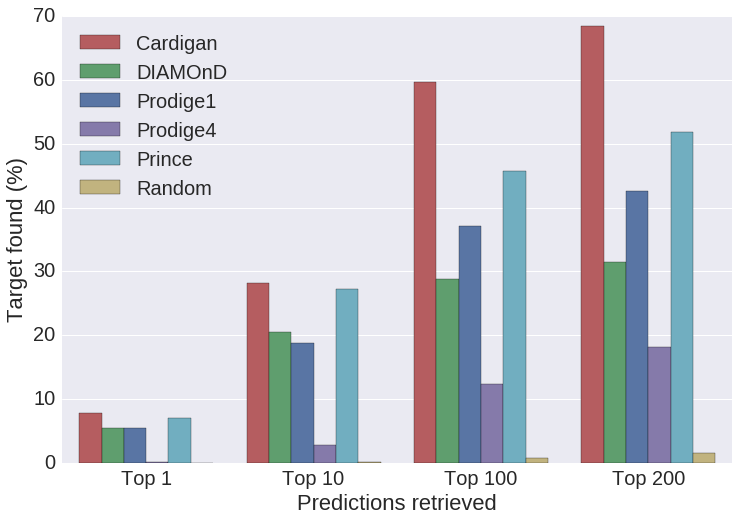


**Fig I. Leave-one-out Charted using DiamondNet.** The 2017 OMIM database contains 264 diseases with two or more genes, which result in 970 possible test cases, 875 of them can be predicted using DiamondNet.


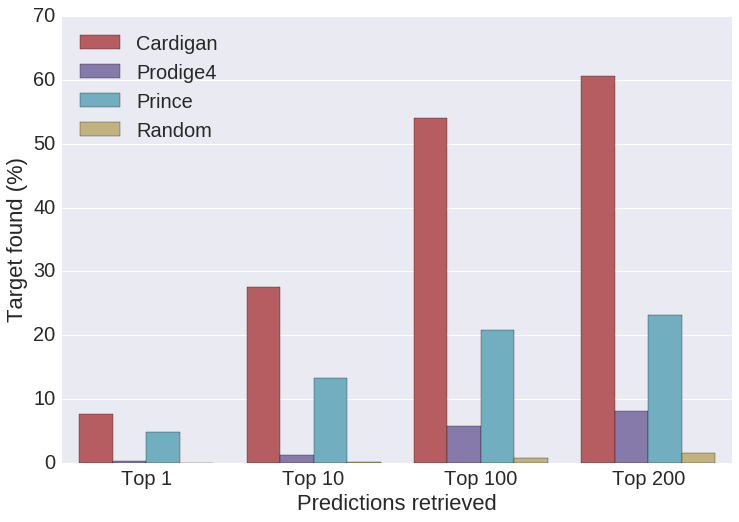


**Fig J. Leave-one-out Uncharted using DiamondNet.** There are 4577 diseases with a single disease gene in the 2013 OMIM database, which result in 4577 possible test cases, 3029 of them can be predicted using DiamondNet.

### Results using BioGRID


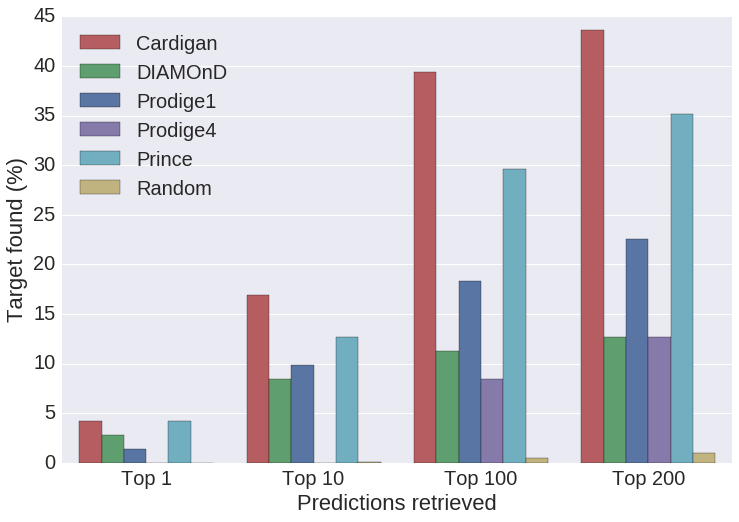


**Fig K. Time Lapse Charted using BioGRID.** Out of the 1413 disease gene associations which were new in the 2017 version of OMIM, only 95 of them were added to diseases which were already charted in 2013, of which 71 can be predicted using BioGRID.


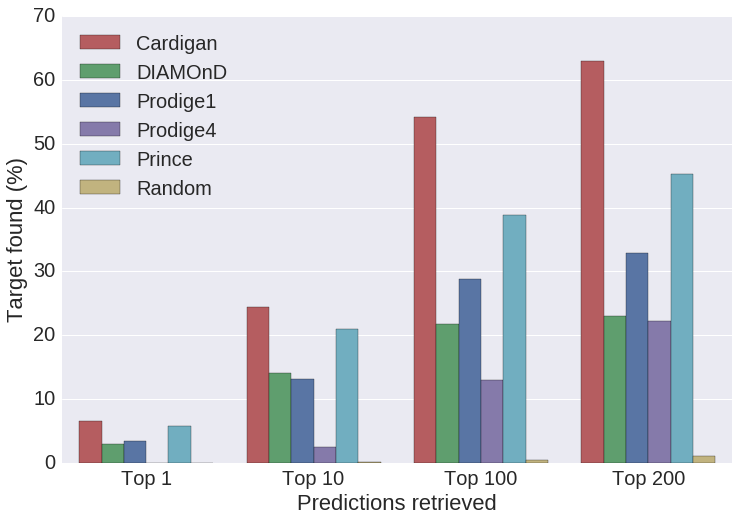


**Fig L. Leave-one-out Charted using BioGRID.** The 2017 OMIM database contains 264 diseases with two or more genes, which result in 970 possible test cases, 893 of them can be predicted using BioGRID.


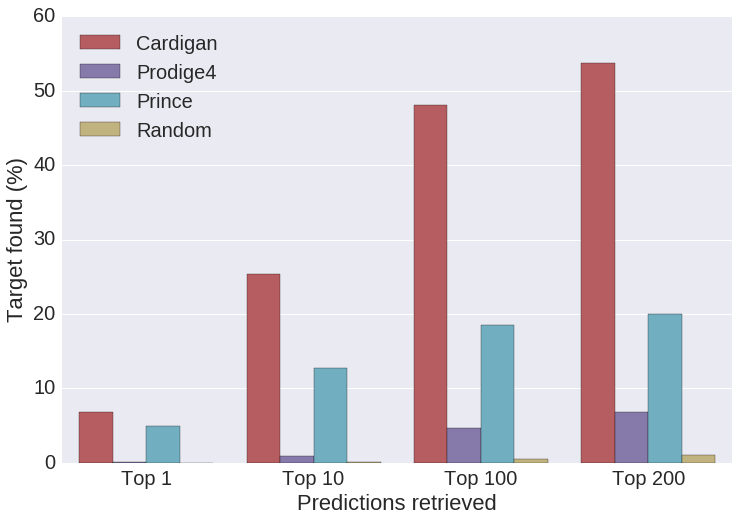


**Fig M. Leave-one-out Uncharted using BioGRID.** There are 4577 diseases with a single disease gene in the 2013 OMIM database, which result in 4577 possible test cases, 3208 of them can be predicted using BioGRID.

### Results using HIPPIE


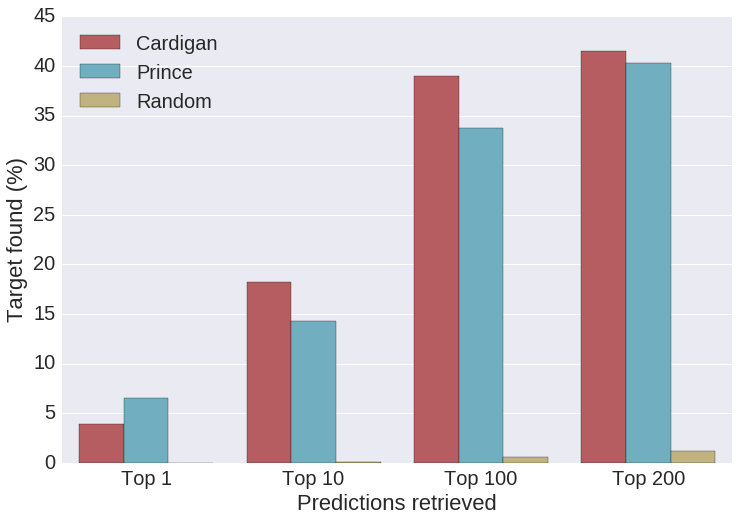


**Fig N. Time Lapse Charted using HIPPIE.** Out of the 1413 disease gene associations which were new in the 2017 version of OMIM, only 95 of them were added to diseases which were already charted in 2013, of which 77 can be predicted using HIPPIE.


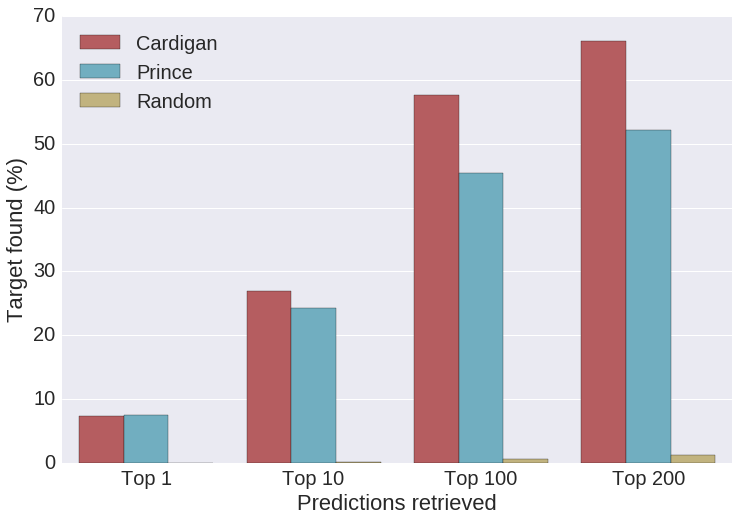


**Fig O. Leave-one-out Charted using HIPPIE.** The 2017 OMIM database contains 264 diseases with two or more genes, which result in 970 possible test cases, 932 of them can be predicted using HIPPIE.


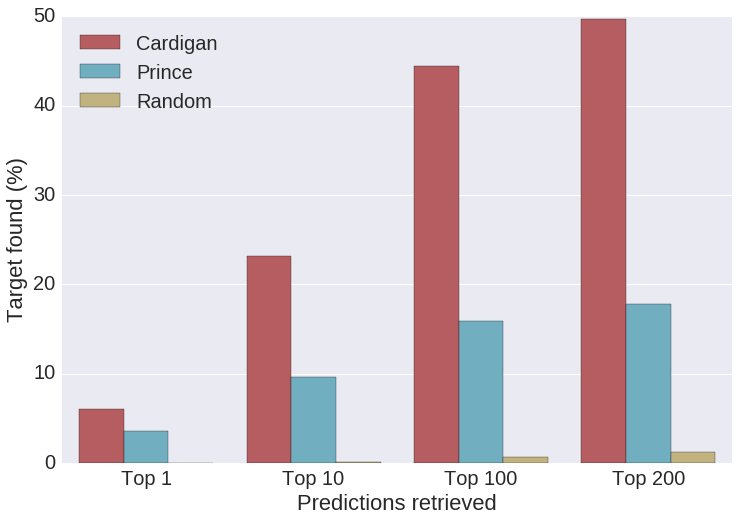


**Fig P. Leave-one-out Uncharted using HIPPIE.** There are 5707 diseases with a single disease gene in the 2017 OMIM database, which result in 5707 possible test cases, 4518 of them can be predicted using HIPPIE.

### Results using FUNCOUP


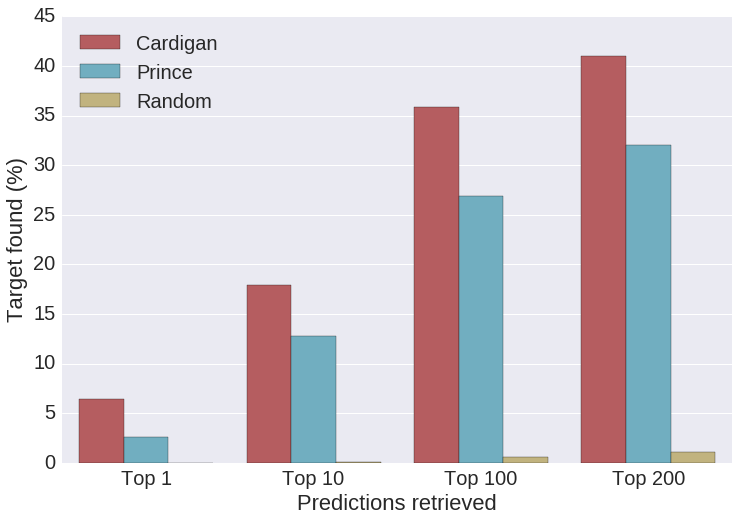


**Fig Q. Time Lapse Charted using FUNCOUP.** Out of the 1413 disease gene associations which were new in the 2017 version of OMIM, only 95 of them were added to diseases which were already charted in 2013, of which 78 can be predicted using FUNCOUP.


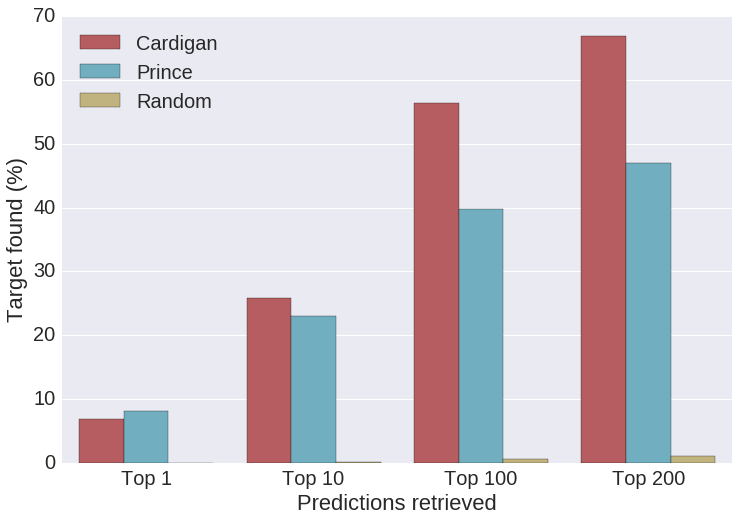


**Fig R. Leave-one-out Charted using FUNCOUP.** The 2017 OMIM database contains 264 diseases with two or more genes, which result in 970 possible test cases, 924 of them can be predicted using FUNCOUP.


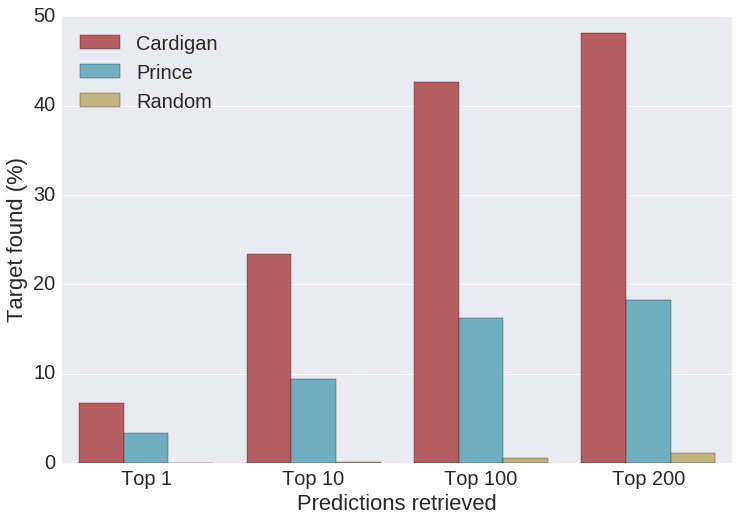


**Fig S. Leave-one-out Uncharted using FUNCOUP.** There are 5707 diseases with a single disease gene in the 2017 OMIM database, which result in 5707 possible test cases, 4543 of them can be predicted using FUNCOUP.

## Running the code

### Dependencies

We provide the code to run Cardigan on Python 2.7, however the code has the following library dependencies:

- NumPy http://www.numpy.org
- SciPy https://www.scipy.org

Besides that, there are some data dependencies to run the code, however terms of service prevent us from the redistribution of most of the following datasets:

- OMIM database: the disease-gene association file (morbidmap), and the MIM to gene translation file (mim2gene).
- Caniza matrix: the code to generate this matrix is provided by the authors http://www.paccanarolab.org/disease_similarity/, and we host the 2017 computed matrix used for this paper.
- PPIs: we provide parsers for the original networks used in the paper (HPRD, DiamondNet, BioGRID, HIPPIE, FUNCOUP). We use Entrez as our preferential identifier, when this identifier is not available in the database, we require an extra translation file to obtain the Entrez identifier.

### Installation

Download the desired bundle from our website http://www.paccanarolab.org/cardigan/, and decompress it in the target directory. Further instructions can be found in the README file.

### Example

To use our software you should import the Cardigan module from our project. The primary class within the module is also called Cardigan. Gene predictions can be made using:

***The predict function***

| cardigan.predict(diseaseMims, seedGenes='default', targetGenes=[]) |
| --- |

The basic prediction using Cardigan can be done in a single line (see Minimum working example). This prediction uses all the disease-gene associations found in the provided OMIM database.

***Minimum working example***

| import Cardigan  # load the module using the default configuration  cardigan = Cardigan.Cardigan()  # predict genes for BDPLT16 (MIM: 187800)  out = cardigan.predict(['187800'])  print out |
| --- |

However you can modify the parameters of the predict function to run a synthetic *leave-one-out* test.

***Example: Synthetic leave-one-out for a charted disease***

| import Cardigan  # load the module using the default configuration  cardigan = Cardigan.Cardigan()  # predict genes for BDPLT16 (MIM: 187800)  # keep gene 3690 as a seed and use 3674 as a target  out = cardigan.predict(['187800'],['3690'],['3674'])  targetPos = cardigan.evaluate()  print targetPos |
| --- |

Simple working examples are provided in the bin/ folder of our software package. Further details can be found in the README file.

## Relation between Cardigan and the Lippert method

Lippert *et al.* ^4^ propose to use the Maximum Mean Discrepancy (MMD) between a sample and a set of known genes to produce a ranking and predict synthetic lethality. However, their method can be used to produce disease gene predictions by changing the input data.

This method empirically computes the MMD of distributions $X=\left\{x_{1},\ldots,x_{m}\right\}$ and $Y=\left\{y_{1},\ldots,y_{n}\right\}$ with a kernel function $k(x,y)=\varphi(x)^{T}\varphi(y)$ according to:

$$\begin{matrix} MMD(k,X,Y) & =\left\|\frac{1}{m}\sum_{i=1}^{m} \varphi(x_{i})-\frac{1}{n}\sum_{i=1}^{n} \varphi(y_{i})\right\| \\ & =\left[\frac{1}{m^{2}}\sum_{i,j=1}^{m} k(x_{i},x_{j})-\frac{1}{mn}\sum_{i,j=1}^{m,n} k(x_{i},y_{j})+\frac{1}{n^{2}}\sum_{i,j=1}^{n} k(y_{i},y_{j})\right]^{\frac{1}{2}} \end{matrix}$$

In practice, the method calculates the negative squared MMD of a particular gene $x\notin D_{C}$ and a set of known genes $D_{C}$, according to:

$$score(x)=-\left\|\varphi(x)-\frac{1}{\left|D_{C}\right|}\sum_{x_{i}\in D_{C}} \varphi(x_{i})\right\|^{2}$$

The ranking is given by sorting the scores of all genes outside of $D_{C}$.

While both our and Lippert’s approaches produce a ranking using a diffusion method, there is an important difference that makes our soft labelling not naturally applicable to their method. The Lippert method starts with a set of known diseases gene, $D_{C}$. In this setup, a gene is either a diseases gene or it is not, that is, the membership to $D_{C}$ is binary. Our idea here was to use soft labels and allow non-binary memberships, that is, all disease genes can be regarded as disease genes for a certain disease to a certain degree.

Note that the scoring function establishes a partition between the possible candidates $x$ and the target set $D_{C}$. In order to use our QWS, we would need to establish a cut-off threshold since $D_{C}$ is not a fuzzy set. Furthermore, when scoring the results, it is unclear whether genes with a high label value which were included as members of $D_{C}$ should be considered as possible predictions or not. We believe that it should be possible to develop an extension of the Lippert et al method to accommodate this, but we feel that it is outside the scope of this work.

## Generalization of Cardigan as a methodology to include soft labels

We believe that our approach to use weighted initial labels can be interpreted as an algorithmic scheme, and extended to use other kernels and ranking functions. Similar ideas are explored by Valentini *et al.* ^5^ in a tool (RANKS) that generalizes and integrates kernelized gene scoring functions to rank putative disease genes.

From the point of view of the RANKS framework, Cardigan proposes to decouple the initial labels from the known genes. The original RANKS framework takes a kernel $K(x,y)$, where $x$ and $y$ are genes, and a set of known disease genes $V_{C}$, which are used to score other genes in the network. For instance, the *average score* for gene $v$ is:

$$S_{avg}(v,V_{C})=\frac{1}{\left|V_{C}\right|}\sum_{x\in V_{C}} K(v,x)$$

We can obtain the same score by slightly changing the above equation and introducing a binary vector $Y(x)=1$ for every gene in $V_{C}$ and 0 otherwise. The *average score* for gene $v$ can be written as:

$$S_{avg}(v,Y,V_{C})=\frac{1}{\left|V_{C}\right|}\sum_{x=1}^{n} K(v,x)Y(x)$$

Cardigan can then be formulated within the RANKS framework in the following way. The closed form of the Zhou diffusion $F=\beta\left(I-\alpha S\right)^{-1}Y$ can be split into the kernel $K=\beta\left(I-\alpha S\right)^{-1}$ , the annotations $Y$(the QWS) and the known genes $V_{C}$. Then, the Cardigan scoring $S_{Cardigan}$ can be defined as:

$$S_{Cardigan}(v,Y,V_{C})=\sum_{x=1}^{n} K(v,x)Y(x)$$

Notice that regardless of the values in the vector $Y$, only genes included in $V_{C}$ should not be scored as possible predictions. With the soft labelling, many genes in $Y$ get a non-zero value, and are still considered for the prediction.

Following this notation, it is clear that our approach fits a generalized framework to include soft labels (the QWS) in kernelized gene scoring techniques. This generalization can be also seen as an extension to the RANKS framework.

# Additional captions

**Supplementary Data 1. Ghiassian Disease dataset to OMIM identifier mapping.** The diseases used in the DIAMOnD paper are not necessarily OMIM diseases, so we manually mapped them to OMIM diseases by matching OMIM disease names and taking into account their description

**Supplementary Data 2. Cardigan prediction on the entire 2017 OMIM dataset.** This is a tab separated file containing disease gene predictions for all the diseases with at least one associated paper in the OMIM database.

# References

1 Zhou, D., Bousquet, O., Navin Lal, T., Weston, J. & Schölkopf, B. Learning with Local and Global Consistency. *Advances in Neural Information Processing Systems* **16**, 321-328 (2004).

2 Bishop, C. M. Pattern recognition and machine learning (information science and statistics) springer-verlag new york. *Inc. Secaucus, NJ, USA* (2006).

3 Goh, K. I. *et al.* The human disease network. *Proc Natl Acad Sci U S A* **104**, doi:10.1073/pnas.0701361104 (2007).

4 Lippert, C., Ghahramani, Z. & Borgwardt, K. M. Gene function prediction from synthetic lethality networks via ranking on demand. *Bioinformatics* **26**, 912-918 (2010).

5 Valentini, G. *et al.* RANKS: a flexible tool for node label ranking and classification in biological networks. *Bioinformatics* **32**, 2872-2874 (2016).

1. While ProDiGe4 could produce results for the entire network, it failed to retrieve any predictions among the top 200 results when the target gene was outside of the main connected component. [↑](#footnote-ref-1)
